# MR arthrography: correlation between anatomic intraarticular variants of the long head of the biceps tendon (long head biceps tendon) and superior labral anterior to posterior (SLAP) lesions

**DOI:** 10.1186/s10195-022-00631-0

**Published:** 2022-03-08

**Authors:** Marco Calvi, Maria Chiara Morgano, Nicola Tarallo, Giuseppe Basile, Giorgio Maria Calori, Leonardo Callegari, Eugenio Annibale Genovese

**Affiliations:** 1 Department of Diagnostic and Interventional Radiology, University of Insubria, Varese, Italy; 2 Department of Radiology ASST Valle Olona, Busto Arsizio Hospital, Busto Arsizio, Italy; 3 Department of Diagnostic and Interventional Radiology, ASST-Settelaghi, Ospedale di Circolo e Fondazione Macchi, Varese, Italy; 4 Trauma Surgery IRCCS Orthopaedic Institute Galeazzi, Milano, Italy; 5 Department of Reconstructive and Prothesic-Revision Surgery- Sepsis, San Gaudenzio Clinic - High Speciality Institute, Novara, Italy; 6 Medical Clinical Institute Intermedica - Columbus, Milano, Italy

**Keywords:** Shoulder, Anatomical variants, SLAP lesions, Long head biceps, MR arthrography

## Abstract

**Background:**

The purpose of this work is to characterize the anatomy of the intraarticular portion of the long head of the biceps tendon (long head biceps tendon) using magnetic resonance (MR) arthrography by investigating whether anatomical variants may facilitate the onset of a supraequatorial lesion (superior labral anterior to posterior, SLAP).

**Materials and methods:**

In 482 shoulder MR arthrographies, we considered the anatomical variants of the intraarticular portion of the long head of the biceps tendon classified according to Dierickx’s arthroscopic classification; lesions of supraequatorial structures were considered in the data analysis. For each anatomical variant, correlation with SLAP and the odd ratio were statistically evaluated, using Fisher’s exact (or chi-squared) test and logistic regression analysis, respectively.

**Results:**

In the mesotenon-type variant, the SLAP frequency was higher than expected [*χ*^2^ (df = 4) = 14.9, *p* = 0.005] with a higher risk of developing a type I SLAP (*p* = 0.0003). In the adherent-type variant, the type II SLAP frequency was higher than expected [*χ*^2^ (df = 3) = 18.1, *p* = 0.0004] with a higher risk of developing type II SLAP (*p* = 0.0001). Two cases of “split” (SPL) long head biceps tendon had III and type IV SLAP, respectively. These patients have a higher risk for type IV SLAP [odds ratio (OR) 19.562, 95% confidence interval (CI) 1.604–238.541, *p* = 0.001]. An increased risk of developing SLAP type II was calculated for male subjects (OR 3.479, 95% CI 1.013–11.951, *p* = 0.019).

**Conclusions:**

It is possible that adherence of the long head biceps tendon to the supraspinatus more often predisposes to a lesion of the superior glenoid labrum (SLAP), in view of the close relationships between the fibrocartilage and the bicipital anchor, probably related to the limited excursion of the intraarticular long head biceps tendon.

## Highlights

- Anatomical variants of the intraarticular portion of the long head of the biceps tendon should be studied with MR arthrography.
- Some anatomical variants are more frequently associated with supraequatorial lesions.
- The higher frequency of lesions in the presence of certain anatomical variants is probably related to the limited excursion of the intraarticular long head biceps tendon.

## Introduction

### Anatomical background

The upper region of the shoulder is anatomically complex; important structures participate in its stability and proper biomechanical function. Among them, the rotator interval, the coracohumeral ligament (CHL), and the superior glenohumeral ligament (SGHL) are crossed by the intraarticular portion of the long head of the biceps tendon (long head biceps tendon) humeri [1–3].

The CHL and SGHL form a sling-like band surrounding the long head of the biceps tendon proximal to the bicipital groove. This biceps reflection pulley plays a key role in the stability of the intraarticular biceps’ tendon [2]. The rotator interval is a complex triangular anatomic space, located in the anterosuperior aspect of the shoulder, bounded by the coracoid process at the base, superiorly by the anterior margin of the supraspinatus muscle tendon and inferiorly by the superior margin of the subscapularis muscle tendon. It plays a major role in shoulder proprioception as it is the intersection point of numerous active and passive stabilizers [2].

### The importance of magnetic resonance (MR) arthrography

Evaluation of the superior zone of the shoulder is diagnostically challenging from an imaging standpoint because of the considerable number of normal variations that occur in this location and the difficulty of accurately assessing superior labral injuries. MR arthrography appears to be the best imaging technique currently available for the evaluation of the glenohumeral joint [3].

The sensitivity and specificity of MR arthrography are greater than those of magnetic resonance imaging (MRI) in terms of finding pathologies and anatomic variants of the superior region [4–7].

Preoperative MRI without contrast medium has often failed to show some lesions later found arthroscopically. The defect in lesion identification has been explained by the need for capsular distension, which makes possible better visualization of all anatomic structures and greater contrast between the glenoid labrum, capsule, capsular recess, glenohumeral ligaments, and articular surface of the rotator cuff [5, 7–10]. MR arthrography distends the joint and allows better definition of the anatomy [11, 12].

Moreover, SLAP lesions are difficult to diagnose clinically [13–15] and are often diagnosed using MR arthrography [14, 15] before proceeding to arthroscopic evaluation.

### Pathology background

Anatomical variants of the origin of long head biceps tendon are reported with a frequency of 1.9–7.4% [2, 3], and numerous anatomical variants such as complete absence, split or Y variant, or extracapsular origin have also been described [16]. Superior labral tears from anterior to posterior (SLAP) lesions are a common pathology in overhead-throwing athletes. [17–19]. Snyder et al. classified SLAP lesions into four types. Type II SLAP lesions are the most common and involve detachment of both the superior labrum and the biceps anchor from the glenoid [20].

The purpose of this work is to characterize the anatomy of the intraarticular portion of the long head biceps tendon using MR arthrography by investigating whether anatomical variants may facilitate the onset of a SLAP lesion.

## Materials and methods

### Patients and study design

The present investigation was designed as a monocentric, nonrandomized, retrospective study based on anonymized consecutive data.

We retrospectively reviewed 482 shoulder MR arthrographies progressively performed in the radiology department of our hospital from January 2017 to December 2020.

The long head biceps tendon anatomical variants of the intraarticular portion were considered and classified according to Dierickx’s arthroscopic classification (Table 1) [21], on the basis of the relationship with the supraspinatus tendon [2]. Anatomical variants and lesions of supraequatorial structures were considered in the data analysis. Both the relative risk (OR) of developing a supraequatorial lesion (SLAP) in the presence of specific anatomical variants and the actual frequency of each of these variants in the study sample were assessed.

**Table 1:** Anatomical variants of the intraarticular portion course of long head biceps tendon. Dierikx's arthroscopic classification [21]

MESO	The ‘‘mesotenon’’ family contains five types of connections that allow particularly good movement between the long head biceps tendon and the rotator cuff	MESO-VI (vinculum)
		MESO-SB (small band)
		MESO-PU (pulley-like sling)
		MESO-PA (partial mesotenon)
		MESO-CO (complete mesotenon)
ADH	The “adherent” family contains four types of stronger connections between a single long head biceps tendon and the capsule	ADH-PM [partially medially adherent to the supraspinatus (SSP)], ADH-PL (partially laterally adherent to the SSP), ADH-CL (completely adherent; attaching to the labrum)
		ADH-CO (completely adherent to SSP; not attaching to the labrum)
SPL	The “split” family contains the two types of split biceps	SPL-DO (split biceps double origin)
		SPL-RE (split biceps reversed type)
ABS	Indicates patients with a complete absence of long head biceps tendon	ABS (complete absence of long head biceps tendon)

*MESO* mesotenon, *ADH* adherent, *SPL* split, *ABS* absence, *long head biceps tendon* long head of the biceps tendon

### Exclusion criteria

The following exclusion criteria were considered: complete tendon injury of the supraspinatus muscle (91 patients), previous shoulder surgery (33 patients), presence of major motion artifacts or inadequate joint distension by the contrast medium (22 patients), adhesive capsulitis (6 patients), and complete inveterate long head biceps tendon injury (4 patients).

Variants related to intraarticular long head biceps tendon morphology and insertion variants on the humeral glenoid were not considered in our series.

### MR-arthrography imaging protocol

A 1.5-Tesla MR imaging system (Achieva XR, Philips) was used with a dedicated shoulder array coil. The patients were placed supine with the shoulder in neutral position, the arm placed along the side and the thumb pointing upwards. All patients were asked to give written informed consent before the procedure. MR arthrography was performed immediately after the intraarticular injection of 20 ml of paramagnetic contrast medium (Dotarem 2.5 mmol/l, Guebet). The image acquisition protocol is summarized in Table 2.

**Table 2:** Detailed MRI protocols

Multiplanar (coronal, axial, and sagittal plane) T1-weighted spin-echo sequences with isotropic voxel: repetition time (RT) 9.5 ms, echo time (ET) 4.7 ms, flip angle (FA) 7°, matrix 320 × 307 pixels, 0.8 × 0.8 mm pixel size, number of signal averages (NSA) 1, thickness 0.54 mm; RT 500 ms, ET 12 ms, thickness 3.5–4 mm	Oblique coronal and sagittal T1-weighted turbo spin-echo sequences (TSE T1): RT 500 ms, ET 18 ms, FA 90°, matrix 384 × 307 pixels, 0.8 × 0.8 mm pixel size, NSA 1, thickness 3.5–4 mm	Coronal fat-saturated PD/T2-weighted (dual) fast spin-echo sequences (FSE PD/T2 FAT SAT): RT 4000 ms, ET 10/80 ms, FA 90°, matrix 230 × 256 pixels, 0.8 × 0.8 mm pixel size, NSA 1, thickness 3.5–4 mm

The field of view (FOV) was variable from 16 to 20 cm

### Statistical analysis

Statistical analysis was carried out by assessing the correlation between qualitative variables organized in contingency tables, using Fisher’s exact test for 2 × 2 tables and chi-squared test for larger tables. A stepwise logistic regression was then carried out to find significant disease risk factors and their odds ratios. A significance level of 95% (*p* < 0.05) was applied to all statistical analyses.

## Results

In total, 326 MR arthrographies were included in the study, and 156 studies were excluded:

- 91 complete tendon injuries of the supraspinatus muscle
- 33 previous shoulder surgery
- 22 major motion artifacts or inadequate joint distension
- 6 adhesive capsulitis
- 4 complete inveterate long head biceps tendon injury

The selected MR arthrographies were performed in 317 patients, (218 males and 99 females, mean age 46.5 years, age range 18–73 years), with the right shoulder being evaluated in 187 cases and the left in 139.

**Fig. 1:**
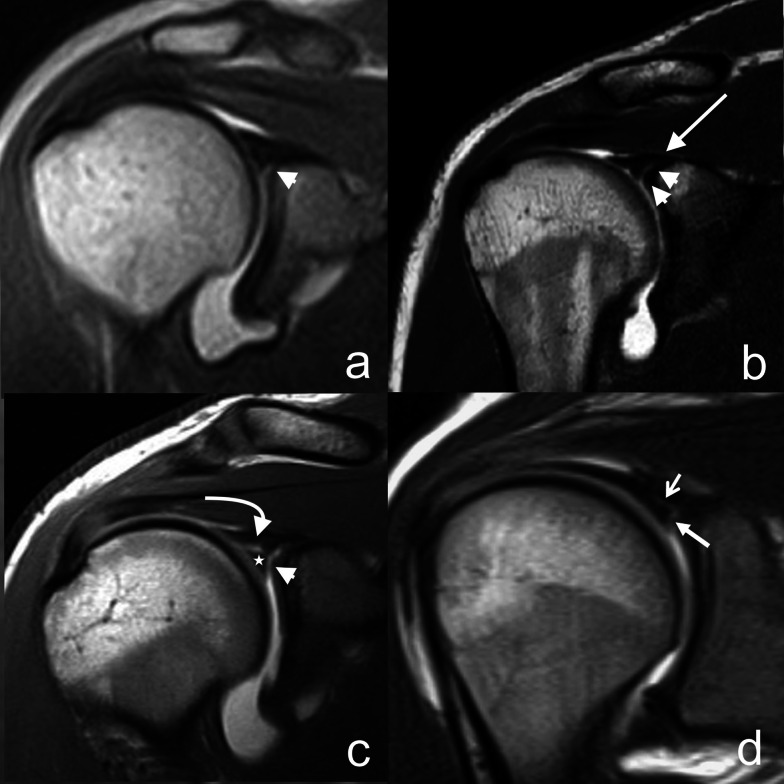
The images in **a**–**d** are all T1-weighted MR arthrographies, acquired in coronal-oblique section after intraarticular injection of approximately 20 ml of paramagnetic contrast agent. In particular, image **a** shows a type I SLAP lesion (arrowhead); image **b** shows a type II SLAP lesion (arrowheads) that does not reach the bicipital anchor (arrow); image **c** displays a type III SLAP lesion where the lesion (arrowhead) extends in a centrifugal direction alongside the bicipital anchor (curved arrow) delimiting a triangular fragment of fibrocartilage (star); image **d** displays a type IV SLAP lesion where the lesion (thick arrow) reaches and interrupts part of the bicipital anchor (thin arrow). **c** and **d** are reprinted with the permission of E. A. Genovese [3]

Using Fisher's and chi-squared tests, we calculated the frequency distribution of SLAP-type lesions (Fig. 1) in the different anatomical variants of the intraarticular course of the long head biceps tendon.

The results are summarized in Tables 3 and 4.

**Table 3:** Association between SLAP lesions and long head biceps tendon variants

	Mesotenon (MESO)	Adherent (ADH)	Split (SPL)
Patients	31/326 (9.5%)	40/326 (12.3%)	3/326 (0.9%)
SLAP absence^a^	25/31 (80.6%)	25/40 (62.5%)	1/3 (33.3%)
SLAP presence^a^	6/31 (19.4%)	15/40 (37.5%)	2/3 (66.7%)

^a^Summary results

**Table 4:** Detailed distribution of SLAP lesions among the long head biceps tendon variant's groups

	MESO-PU	MES-PA	MESO-SB	MESO-VI	ADH-CL	ADH-PL	ADH-PM	SPL-DO	Male	Female
SLAP 1	–	1	2	1	2	1	1	–		
SLAP 2	–	–	–	1	2	–	3	–	88.9%	11.1%
SLAP 3	–	–	–	1	3	1	1	1		
SLAP 4	–	–	–	–	2	–	–	1		

Distribution of patients (*n*)

In the subgroup of the mesotenon-type variant (MESO) [31/326 (9.5%)], 6/31 (19.4%) had a SLAP lesion. Of these, 4/6 had a type I SLAP, 1/6 had a type II SLAP, and 1/6 had a type III SLAP. No patients with the MESO variant had a type IV SLAP lesion.

**Fig. 2:**
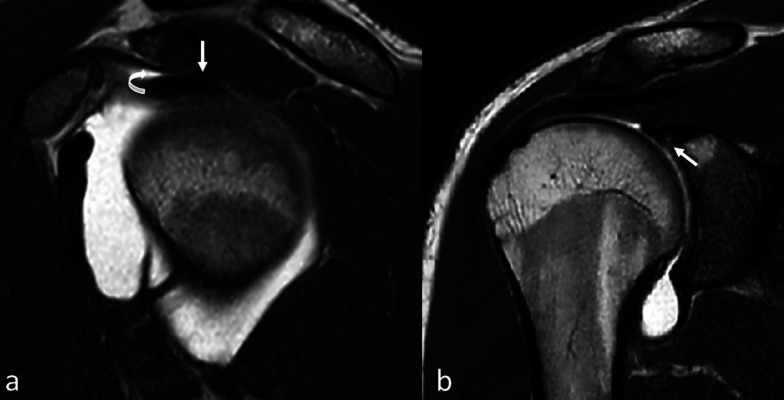
**a** MR arthrography, sagittal SE T1-weighted. In MESO-PA anatomical variant, long head biceps tendon is partially fused to the inferior surface of the supraspinatus tendon (arrow). Contrast agent outlines acute angle between the anterior portion of the long head biceps tendon and the inferior surface of the supraspinatus (curved arrow). **b** Same patients as in **a**. MR arthrography, coronal SE T1-weighted. MESO-PA anatomical variant is associated with SLAP lesion type I. MESO-PA anatomical variant is associated with SLAP lesion type I (arrow)

A higher-than-expected frequency was found in the group of patients with SLAP 1 and MESO-SB [*χ*^2^ (df = 4) = 14.9, *p* = 0.005]. The risk of developing a type I SLAP lesion was higher in the MESO-PA anatomical variant group (*p* = 0.0003) (Fig. 2). The OR of the subgroups could not be evaluated owing to the small number of patients for each group.

In the subgroup with adherent long head biceps tendon (ADH) [40/326 (12.3%)], 15/40 (37.5%) had a SLAP lesion. Of these, 4/15 had a type I SLAP, 5/15 had a type II SLAP, 5/15 had a type III SLAP, and 2/15 had a type IV SLAP.

**Fig. 3:**
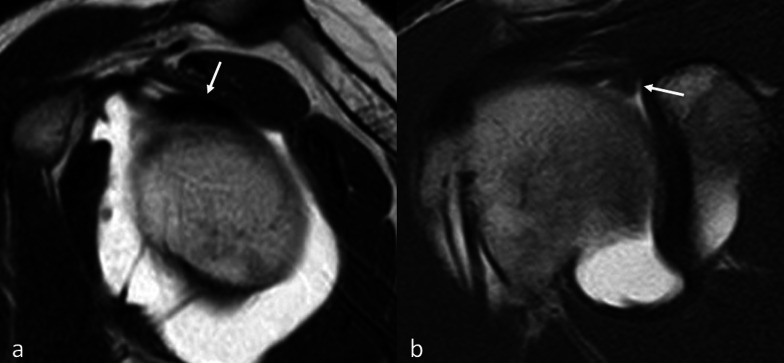
**a** MR arthrography, sagittal SE T1-weighted. In ADH-PM anatomical variant, long head biceps tendon is partially medially to the inferior surface of the supraspinatus (arrow). **b** Same patients as in **a**. MR arthrography, coronal SE T1-weighted. ADH-PM anatomical variant is associated with SLAP lesion type II (arrow)

Compared with expectation, we found a higher frequency of type II SLAP in patients with adherent ADH-PM long head biceps tendon [*χ*^2^ (df = 3) = 18.1, *p* = 0.0004]. In addition, patients with the ADH-PM long head biceps tendon variant also have an increased risk of developing type II SLAP (*p* = 0.0001) (Fig. 3). Again, the small number of subgroups did not allow the OR to be calculated.

**Fig. 4:**
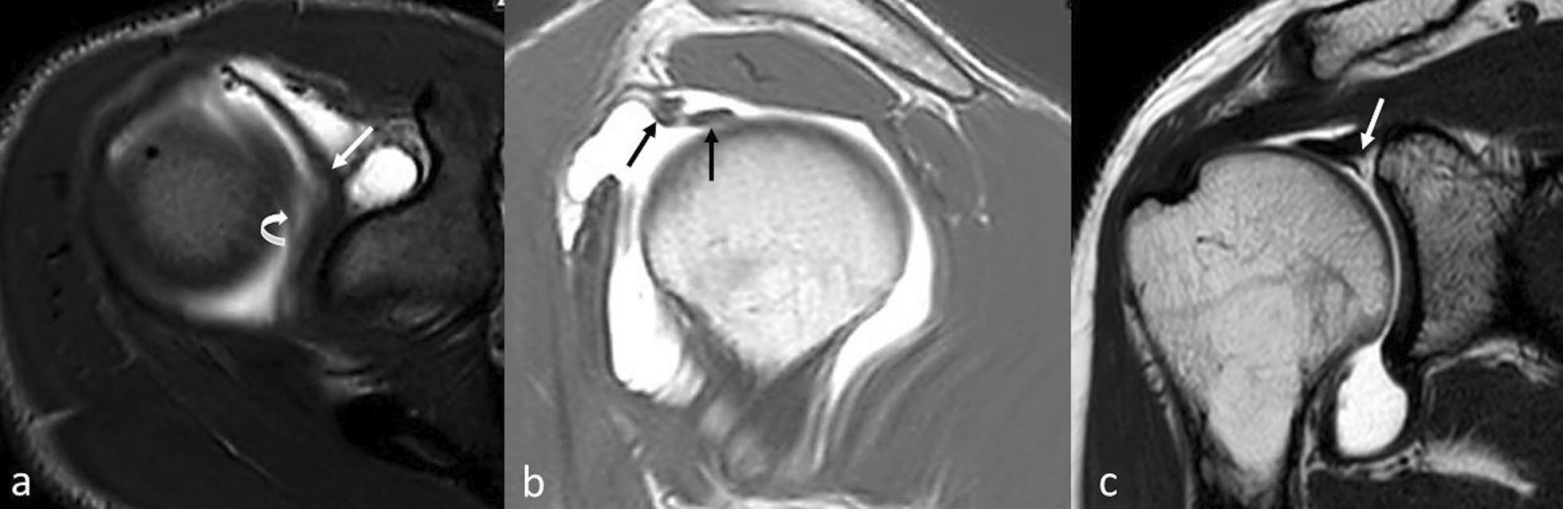
**a** MR arthrography, T1-weighted with fat signal saturation (FAT-SAT) imaging on axial plane. SPL-DO anatomical variant (long head biceps tendon has double band) comes from supraspinatus tendon (white curved arrow) and from glenoid (white arrow). **b** MR arthrography, sagittal SE T1-weighted. SPL-DO anatomical variant (double beams of long head biceps tendon; black arrows). **c** MR-arthrography, coronal SE T1-weighted. SPL-DO anatomical variant is associated with SLAP lesion type IV (white arrow)

Both the cases of split (SPL) long head biceps tendon [2/326 (0.6%)] had SLAP lesions, types III and IV, respectively. We also found an increased risk of developing type IV SLAP in those patients who presented with a split long head biceps tendon as an anatomical variant with OR 19.562 (95% CI 1.604–238.541, *p* = 0.001) (Fig. 4).

Among patients with SLAP type II, 88.9% were male and 11.1% were female; an increased risk of developing SLAP type II was calculated for male subjects, with OR 3.479 (95% CI 1.013–11.951, *p* = 0.019).

## Discussion

The main result emerging from the obtained data is that, indeed, there is a correlation between the presence of specific anatomical variants of the intraarticular portion of the long head biceps tendon and the SLAP lesion. Until now, many of these conditions have been considered insignificant and not responsible for shoulder pathology.

Only a few authors have looked for a relationship between anatomic variants and upper shoulder pathologies in adults [22–24]. Studies in the literature show an association between distinct types of long head biceps tendon anchorage and shoulder instability secondary to post-traumatic biceps anchor injury [14]. In particular, an association between posterior anchorage and SLAP lesion is demonstrated. [14]

Many authors have described pathologies and anatomic variants of the superior region of the shoulder [1, 3, 7, 14, 21, 25–34]. However, few authors classified or analyzed the variants systematically with a large group of participants [21, 25]. Some studies have classified or analyzed the anatomical variants of long head biceps tendon [21], while others have examined the diagnostic accuracy of MRI in diagnosing pathological patterns [35]. However, these studies were mainly conducted in small groups of patients and not systematically performed.

Congenital variants of the relationship of the long head biceps tendon with the rotator cuff have been reported in the literature; some have described absence of long head biceps tendon [22, 30–33], others the intracapsular and extracapsular course of the tendon [22, 23, 36]. Anatomical variants of long head biceps tendon origin are reported to occur with a frequency of 1.9–7.4%.

Our data show that split tendon is a rare finding, found in 0.9% of cases, while the variant characterized by adherent long head biceps tendon with the supraspinatus was seen in 12.3% of cases, followed by the variant with the mesotenon, in 9.5% of cases.

Many studies have shown that these conditions are inherited and are the result of partial detachment from the mesothelium or synovial fusion with the inferior surface of the capsule [37–39].

With respect to the association between certain anatomic variants and the presence of SLAP lesions, in our case series, it appears that the long head biceps tendon mesotenon (MESO-PA) variant may predispose to superficial shoulder pathology; specifically, we found a higher-than-expected frequency of SLAP 1 in the group of patients with MESO-PA anatomical variant. The OR for this variant, though, could not be calculated.

To the best of our knowledge, there are no studies in the literature that can confirm or disprove this finding. The reason for this apparent association could be found in a decreased range of movement of the intraarticular portion of the long head biceps tendon.

In concordance with the literature, we found significant correlation between the adherent long head biceps tendon group and the free long head biceps tendon group for SLAP lesion [12, 13, 40, 41]. In fact, compared with expectation, we found a higher frequency of type II SLAP in patients with adherent (ADH-PM) long head biceps tendon.

This result could also be in line with the association of type I SLAPs in the presence of mesotenon-type anatomic variants. In this case, because there is an additional limitation to the range of motion of the long head biceps tendon intraarticular portion, the association is with a type II SLAP. As the degree of fixation increases, the degree of injury to the glenoid fibrocartilage seems to be higher.

Finally, we found an increased risk of developing SLAP type IV in those patients who presented with a SPL-long head biceps tendon as an anatomical variant. In this case, the presence of this anatomical variant seems to act as a risk factor itself in developing a type IV SLAP lesion.

Again, to the best of our knowledge, there are no studies in the literature that can confirm or debunk this observation. The reasons for this result would appear to belong to a different order than the previously described variants and are likely to be due mainly to biomechanical reasons similar to those leading to a greater association of SLAP in cases of posterior CLB tendon insertion described by Jakanani et al. [14].

In our case series, we did not find any cases of long head biceps tendon agenesis. Four anatomical variants, from hypoplasia of the biceps to its complete absence, have been described in the literature [31]. Other authors have described three variants of the proximal extracapsular portion of the long head biceps tendon [42]. Cases of split tendon and complete fusion with the inferior surface of the rotator cuff have been described in three cases [24, 34, 37].

The strengths of our study are the large initial sample size, the systematic use of MR arthrography, and the single-center design of the study. Other authors evaluated the sensitivity and specificity of MRI versus MR arthrography in 150 shoulders. The sensitivity and specificity values of MRI without contrast medium were respectively 83% and 100% for anterior labrum lesions, 85% and 100% for SLAP lesions, and 92% and 100% for supraspinatus tendon lesions.

Compared to arthroscopy, MR arthrography reaches sensitivity and specificity values respectively of 98% and 100% for anterior labral lesions, specifically 98% and 99% for SLAP lesions and 100% sensitivity and specificity for cuff lesions [7, 13].

A limitation of our study is the absence of systematic comparison between MR-arthrography and arthroscopy, but data in the literature consider MR arthrography the gold standard in the evaluation of the upper portion of the shoulder [5, 43].

## Conclusions

Our findings suggest that the diagnosis of anatomical variants of upper shoulder structures should be performed with MR arthrography.

It is possible that adherence of the long head biceps tendon to the supraspinatus more often predisposes to a lesion of the superior glenoid labrum (SLAP), in view of the close relationships between the fibrocartilage and the bicipital anchor, probably related to the limited excursion of the intraarticular long head biceps tendon.

## Data Availability

The datasets used and/or analyzed during the current study are available from the corresponding author on reasonable request.

## Abbreviations

SLAP: Superior labral anterior to posterior
long head biceps tendon: Long head of the biceps tendon
MESO-VI: Mesotenon variant with vinculum
MESO-SB: Mesotenon variant with small band
MESO-PU: Mesotenon variant with pulley-like sling
MESO-PA: Partial mesotenon
MESO-CO: Complete mesotenon
ADH-PM: Partially medially adherent to the supraspinatus
ADH-PL: Partially laterally adherent to the supraspinatus
ADH-CL: Completely adherent to the supraspinatus attaching to the labrum
ADH-CO: Completely adherent to supraspinatus not attaching to the labrum
SPL-DO: Split biceps double origin
SPL-RE: Split biceps reversed type
ABS: Absence of tendon
